# Isolation of Fucoxanthin from *Sargassum oligocystum* Montagne, 1845 Seaweed in Vietnam and Its Neuroprotective Activity

**DOI:** 10.3390/biomedicines11082310

**Published:** 2023-08-19

**Authors:** Dang Diem Hong, Le Thi Thom, Nguyen Cam Ha, Ngo Thi Hoai Thu, Hoang Thi Minh Hien, Luu Thi Tam, Nguyen Manh Dat, Tran Mai Duc, Nguyen Van Tru, Nguyen Thi Minh Hang, Ranga Rao Ambati

**Affiliations:** 1 Institute of Biotechnology, Vietnam Academy of Science and Technology, 18 Hoang Quoc Viet Str., Cau Giay, Hanoi 100000, Vietnam; thomcnsh@gmail.com (L.T.T.); hanguyen.hou@gmail.com (N.C.H.); nhoaithu2002@yahoo.com (N.T.H.T.); hmhien@ibt.ac.vn (H.T.M.H.); tamluu5458kl@gmail.com (L.T.T.); mdnguyen11102000@gmail.com (N.M.D.); nvtru@ibt.ac.vn (N.V.T.); 2 Department of Microbiology, Graduate University of Science and Technology, Vietnam Academy of Science and Technology, 18 Hoang Quoc Viet Str., Cau Giay, Hanoi 100000, Vietnam; 3 Nha Trang Institute of Technology Research and Application, Vietnam Academy of Science and Technology, Nha Trang 57100, Vietnam; ductranmai@gmail.com; 4 Institute of Marine Biochemistry, Vietnam Academy of Science and Technology, 18 Hoang Quoc Viet Str., Cau Giay, Hanoi 100000, Vietnam; minhhang@imbc.vast.vn; 5 Department of Biotechnology, School of Biotechnology and Pharmaceutical Sciences, Vignan’s Foundation for Science, Technology and Research (Deemed to Be University), Vadlamudi, Guntur 522213, India; arangarao99@gmail.com

**Keywords:** Alzheimer’s, acetylcholinesterase inhibitory, β-amyloid protein fragment, fucoxanthin, health food for humans, *Sargassum oligocystum*

## Abstract

Fucoxanthin extracted and purified from Vietnamese *Sargassum oligocystum* Montagne, 1845 exhibits various biological activities. In this study, the ability of fucoxanthin to inhibit acetylcholinesterase (AChE), the antioxidant activities, and the expression of antioxidant enzymes were investigated. Fucoxanthin isolated from Vietnamese *S. oligocystum* showed no cytotoxic effects; moreover, it exhibited AChE inhibitory activity (with an IC_50_ value of 130.12 ± 6.65 μg mL^−1^) and antioxidant activity (with an IC_50_ value of 3.42 ± 0.15 mg mL^−1^). At concentrations of 50 and 100 µg mL^−1^, fucoxanthin provided protection against amyloid β-protein fragment 25–35-induced neurotoxicity in a C6 neuronal cell line, and the survival of C6 cells was higher than 81.01% and 80.98%, respectively, compared to the control group (59%). Moreover, antioxidant enzyme activity and quantitative PCR analysis suggested that the neuroprotective effect of fucoxanthin resulted from regulation of the gene expression of antioxidant enzymes (CAT and GPx) and ER pathways (caspase-3 and Bax), as well as the promotion of expression of genes involved in PI3K/Akt signaling (GSK-3β), autophagy (p62 and ATG5), and the biosynthesis of ACh (VAChT and ChAT). Therefore, fucoxanthin extracted from the seaweed *S. oligocystum* in Vietnam is a potential feedstock source for the production of health foods that exert neuroprotective effects.

## 1. Introduction

Seaweeds are important marine organisms worldwide, with a diverse range of species that provide high nutritional and health benefits [1]. In recent decades, seaweeds have been cultivated and harvested for the food industry. Fifty countries were actively involved in seaweed farming in 2018, accounting for 97.1% (by volume) of cultivated and harvested seaweed (32.4 million tons total) [2,3].

Brown, red, and green seaweeds contain many bioactive compounds that are widely utilized in agriculture, food, and medicinal applications [1,4,5,6]. Fucoxanthin is found in many seaweed species, including *Undaria*, *Laminaria*, *Eisenia*, *Sargassum*, *Dictyota*, *Fucus*, *and Myagropsis*, as well as in microalgae, such as *Phaeodactylum*, *Cylindrotheca*, *Isochrysis*, *Cyclotella*, *Nitzschia*, *Prymnesium*, *Chaetoceros*, and *Odontella* [7]. Carotenoids, such as astaxanthin and fucoxanthin, are promising nutritional, pharmacological, and medicinal constituents of human health foods [6,8].

Alzheimer’s disease (AD) is the most common form of dementia syndrome and often occurs in elderly individuals. The number of patients with AD is increasing because the average life expectancy is increasing. The rate of new cases has increased by 5–7 million per year, and an estimated 115 million people will develop AD by 2050 [9]. AD is identified as the sixth leading cause of death in the United States, and with high treatment costs, AD places a burden on society [10].

Therefore, researching and developing drugs to treat AD is of interest to researchers worldwide. During the drug research and development phase, extracts/compounds derived from medicinal plants and seaweed play a very important role in the treatment of AD, as half of the drugs licensed for the treatment of AD today are derived from medicinal plants [11]. Drugs derived from medicinal herbs exhibit several advantages, including few side effects and economic benefits for patients with chronic AD [12]. For example, the publication of Aljubiri et al. [13] showed that the extracts of *Euphorbia schimperiana* and *Euphorbia balsamifera* and its purified compounds, such as 4-O-ethylgallic acid and isoorientin, had acetylcholinesterase and tyrosinase inhibition activities, suggesting these extracts and compounds could be used as potential active ingredients of several drugs for neuroprotective effects. Research and development of drugs derived from medicinal herbs in the treatment of AD is a suitable direction in countries with abundant plant and seaweed sources, such as Vietnam.

The excessive generation of free radicals and reduced level of antioxidants in the body leads to an increased risk of cell damage and causes many diseases, such as diabetes, atherosclerosis, inflammatory disorders, and several neurological diseases [14]. However, natural antioxidant compounds can prevent these effects [15,16]. Recently, a marine carotenoid pigment from seaweeds was shown to exhibit potential anti-obesity, antioxidant, and anti-inflammatory activities and reduced neurotoxicity induced by amyloid β protein fragment 1–42 (Aβ_1–42_) [17].

One factor involved in mediating the cellular antioxidant mechanism is nuclear factor erythroid 2-related Factor 2 (Nrf2), a transcription factor that binds to the ARE (Antioxidant Responsive Element) and promotes the redox state of the cell under harmful stress [18]. Under normal conditions, Nrf2 binds to Kelch-like ECH-binding protein 1 (Keap1) and is located in the cytoplasm [19]. However, under oxidative stress, Nrf2 dissociates from Keap1, moves to the nucleus, binds to the ARE, and regulates the expression of antioxidant genes, including heme oxygenase-1 (HO1) and NAD(P)H (nicotinamide adenine dinucleotide phosphate): quinone oxidoreductase (NQO1). A recent study showed that carotenoids suppress the expression of Nrf2, resulting in inhibited apoptosis and oxidative stress [20]. Moreover, carotenoid-loaded poly lactide-co-glycolide with polyethylene glycol (PLGA-PEG) nanoparticles induced antioxidant effects by enhancing the activity of superoxide dismutase (SOD) and catalase (CAT) in the brain [21]. Carotenoids have been shown to protect cells by indirectly upregulating Nrf2 through activating the phosphatidylinositol 3-kinase (PI3K)/protein kinase B (Akt) signaling pathway. Zheng et al. [22] reported that carotenoids elevated the levels of phosphorylated Akt and Nrf2 in human keratinocytes. Furthermore, a specific inhibitor of Akt, LY294002 (2-(4-morpholinyl)-8-phenyl-chromone), significantly suppressed the active form of Akt, which resulted in a reduction in Nrf2 accumulation [22]. However, the mechanism that regulates the Nrf2/ARE pathway and underlies the protective effects of fucoxanthin has not been elucidated and fully explained.

Moreover, the relationship between the metabolism of Aβ oligomers and the PI3K/Akt signaling pathway has been reported by various authors [23,24,25]. Aβ accumulation inhibits PI3K/Akt signaling and increases the activity of glycogen synthase kinase-3β (GSK-3β), a key factor in the occurrence and development of AD. Once GSK-3β is activated, it upregulates tau protein phosphorylation, inducing apoptotic signals and decreasing the survival capacity of the cells [26,27,28]. Lin et al. [29] indicated that pretreatment with marine carotenoids significantly blocked Aβ oligomer-induced alterations to the PI3K/Akt and ERK pathways in the SH-SY5Y cell line.

Recently, Zhang et al. [30] reported that natural antioxidant compounds activated autophagy and provided neuroprotection in brain injury models. Beclin-1 (Beclin-1 is a subunit of PI3K class complexes) is a key protein in autophagy and is necessary for the recruitment of other Atg proteins during the process of autophagy, and LC3 is essential for autophagy formation. P62 (sequestosome-1/A170/Zeta-interacting protein) is a selective autophagy receptor and serves as a common readout of autophagic activity. It was suggested that marine carotenoids could prevent traumatic brain injury (TBI)-induced oxidative stress and apoptosis by decreasing the expression of p62 and increasing the expression of two important autophagic genes, *Beclin*-1 and *LC3*-II, to activate autophagy [30].

Choline acetyltransferase (ChAT) and vesicle acetylcholine (ACh) transporter (VAChT) are specialized proteins for the synthesis, storage, and hydrolysis of acetylcholine. ChAT is related to the biosynthesis of ACh from acetyl-CoA and choline in the cytoplasm, while VAChT is involved in the uptake of the neurotransmitter into synaptic vesicles [31]. Several diseases, such as AD, occur when the expression of those proteins is altered [32]. Chen et al. [33] reported that carotenoid as astaxanthin treatment in ferrous amyloid buthionine (FAB)-infected mice reduced neuroinflammation by restoring the expression of ChAT in cholinergic neurons of the medical septal (MS) nucleus and cholinergic fibers in the hippocampus CA1 region; as a result, spine loss on hippocampal CA1 pyramidal neurons was recovered, and behavioral deficits were improved in AD rats.

In Vietnam, 827 species of seaweeds have been identified. Of these, there are 412 species of red seaweeds (Rhodophyta), 180 species of green seaweeds (Chlorophyta), 147 species of brown seaweeds (Ochrophyta-Phaeophytaceae), and 88 species of cyanobacteria (Cyanophyta) [34,35,36]. *Sargassum* is a genus of brown seaweed and has great economic value because large amounts of alginate are present in the cell wall. The south-central region is the most diverse zone of Vietnamese seaweeds, as 75% of species were recorded from the survey areas [37]. For a long time, Vietnamese seaweeds have been used as health foods, supplementary feeds for domestic animals, raw materials for industry, biofertilizers, biofuels, and a source of new materials and bioactive substances/extracts [35,36,38]. To date, 73 different species of *Sargassum* have been identified in Vietnam, and some species, including *S. oligocystum* Montagne, 1875, provide great sources for the exploitation of biologically active substances.

Among different carotenoids, fucoxanthin has been the focus of research due to its potential applications as a health food for humans. Fucoxanthin is a substance with a known chemical structure, but its biological activity depends on the extraction source and growing conditions. When large amounts of fucoxanthin are extracted for use as a health food, the extracts may not exhibit a very high purity (greater than 90%), so some other substances may contribute to the biological effects of fucoxanthin.

To date, no publication has examined fucoxanthin isolated from the brown seaweed *S. oligocystum*, its neuroprotective activity, and the molecular mechanism of its neuroprotective activity. Therefore, in this study, we extracted fucoxanthin from *S. oligocystum* seaweed in Vietnam, which has a very high natural abundance in Vietnam, depending on the season. Therefore, isolating fucoxanthin from *Sargassum* seaweed species has practical significance, and exploiting substances with neuroprotective and memory-enhancing properties from the seaweed is highly feasible. In this paper, fucoxanthin was extracted from Vietnamese *S. oligocystum* Montagne, 1875 and purified. Then, its acetylcholinesterase (AChE) inhibitory activity, antioxidant activity, and ability to protect cells from cytotoxicity in a C6 AD cell model induced by H_2_O_2_ or Aβ_25–35_ in vitro were measured to elucidate its neuroprotective ability and molecular mechanism. The obtained results demonstrate that fucoxanthin isolated from *S. oligocystum* in Vietnam can be used in functional food applications for human health benefits.

## 2. Materials and Methods

### 2.1. Collection of Seaweed Samples

Nine species of seaweeds belonging to the genus *Sargassum* collected from Khanh Hoa, Ninh Thuan, and Thua Thien Hue in 2007–2008 and 2022 (March–May) were given scientific names by Dr. Huynh Quang Nang at the Nha Trang Institute of Technology Research and Application (NITRA), Vietnam Academy of Science and Technology (VAST), Nha Trang, Khanh Hoa province, Vietnam, and MS. Tran Mai Duc, NITRA, VAST, Nha Trang, Khanh Hoa province, Vietnam, and are presented in Table 1.

### 2.2. Cell Culture and Treatment

C6 rat glial cells (ATCC, CCL-107™) were obtained from Dr. Duong Hoang Nguyen, Center for Soft Matter and Biological Physics, Center for High Technology Development, VAST. The cells were cultured in Dulbecco’s minimum essential medium (DMEM)/high glucose supplemented with 10% (*v*/*v*) fetal bovine serum (FBS) and 1% (*v*/*v*) penicillin/streptomycin under 5% CO_2_ at 37 °C.

### 2.3. Chemicals

Standard fucoxanthin (F6932; Sigma-Aldrich, Singapore); 5-5′-dithiobis-2-nitrobenzoic acid (DTNB) (Sigma-Aldrich, Singapore), acetylthiocholine iodide (ACTI) (Sigma Aldrich, Singapore), DPPH (2,2-diphenyl-1-picrylhydrazyl, purity 95%, Alfa Aesar, Tokyo, Japan), 3-(4,5-dimethylthiazol-2-yl)-2,5-diphenyltetrazolium bromide (MTT, Invitrogen, Thermo Fisher Scientific, Waltham, MA, USA), Aβ_25–35_ (purity ≥ 97%, No. A.4559; Sigma, Ronkonkoma, NY, USA), galantamine hydrobromide (Sopharma AD, Sofia, Bulgaria), fetal bovine serum (FBS), penicillin and streptomycin (Invitrogen, USA), Dulbecco’s modified Eagle’s medium (DMEM)/high glucose, and other common chemicals were used in this study.

### 2.4. Extraction of Fucoxanthin

The extraction of fucoxanthin was performed according to a procedure described by Haugan et al. [39]. A total of 0.5 g (m_1_) of sample was weighed and mixed with 0.6 mL of 90% acetone, shaken well, and soaked for 10–20 min. The extract was obtained, and the residue was further extracted in 0.6 mL acetone for 10–20 min. This process was repeated until the extract became colorless. n-hexane and water were added to the extract at a ratio of extract:n-hexane:water of 3:1:1 (*v*/*v*/*v*). The mixture was divided into the following phases: the upper phase contained n-hexane, and the lower phase contained acetone and water. The upper phase (n-hexane) was collected, and the lower phase was removed. The upper phase was washed using a mixture of methanol:n-hexane at a ratio of 1:1 (*v*/*v*). Then, the n-hexane phase was collected completely. Rotary evaporation at 70 °C was used to remove the solvent to obtain fucoxanthin (m_2_). The fucoxanthin content was calculated according to the following formula:

%F = m_2_/m_1_ × 100

where m_1_ is the initial biomass mass, and m_2_ is the mass of obtained fucoxanthin.

### 2.5. Preparation of Methanol Extract

*S. oligocystum* (100 g) was extracted 4 times each with 300 mL of methanol (MeOH) at room temperature using an ultrasonic bath (BioBase, ultrasonic power 80 w, ultrasonic frequency 4.7 kHz) for 15 min and then resting for 15 min, with completion of extraction in 2 h. The solvent was evaporated using a rotary evaporator (Ilmvac Laboratory Vacuum Pump System WMS 301p and Rotavapor^®^ R-100 Heating Bath B-491, BUCHI Labortechnik AG, Flawil, St. Gallen, Switzerland) under reduced pressure at 37 °C to obtain 2.04 g of methanol extract.

### 2.6. Isolation of Fucoxanthin

*S. oligocystum* (200 g) was extracted with acetone using sonication at room temperature (4 times, 600 mL of acetone, 30 min each time). The combined extracts were evaporated in vacuo at 37 °C to obtain the total acetone extract (3.78 g), which was subjected to a silica gel column and eluted with an n-hexane/acetone gradient system (0 → 50% acetone, *v*/*v*) to obtain the crude fucoxanthin fraction. The crude fucoxanthin fraction (150 mg) was further purified by a Sephadex LH-20 column using CH_2_Cl_2_/MeOH (1:4, *v*/*v*) as eluents to obtain fucoxanthin (1; 13.8 mg).

### 2.7. Column Chromatography Method

Fucoxanthin (1) was isolated using a silica gel 60 column with a particle size of 0.040–0.063 mm (230–400 mesh) from Merck and Sephadex LH-20 (Sigma) and eluted with a mixture of n-hexane/acetone and dichloromethane/methanol solvents.

### 2.8. Thin-Layer Chromatography (TLC)

Crude fucoxanthin was separated on a 10 × 8 cm TLC column (Merck precoated silica gel 60 F254 aluminum sheets, layer thickness 0.2 mm). The mobile phase consisted of chloroform:methanol at a ratio of 98:2 (*v*/*v*). The bands on the chromatographic plate were detected by spraying with 10% H_2_SO_4_ solution and drying on an alcohol lamp.

### 2.9. Determination of Fucoxanthin Content and Purity

Fucoxanthin content and purity were analyzed by high-performance liquid chromatography (HPLC; Shimadzu LC 10ADVP, Kyoto, Japan). The system is equipped with a Symmetry^®^ C18, 5 µm, 100 A° Column (4.6 mm × 250 mm Column); Part Number: WAT054275, made in Ireland. The mobile phase comprised acetonitrile/methanol (1:9, *v*/*v*) at a flow rate of 0.5 mL min^−1^. Fucoxanthin was detected using a PDA probe (Shimadzu, Tokyo, Japan) at 450 nm. The fucoxanthin concentration of the samples was identified by comparing the retention time against a known standard for fucoxanthin.

### 2.10. Determination of Fucoxanthin Structure

The structure of fucoxanthin was determined by nuclear magnetic resonance (NMR) spectra recorded on a Bruker Avance Neo 600 MHz spectrometer (Bruker, Coventry, UK), with CDCl_3_ as the solvent and TMS as the internal standard. ^1^H NMR spectra were measured at 600 MHz, and ^13^C was measured at 150 MHz at the Institute of Chemistry, VAST. The structure of fucoxanthin was determined by comparing the NMR data with the published standard [40].

### 2.11. DPPH Assay

A 2,2-diphenyl-1-picrylhydrazyl (DPPH) assay was performed, and the scavenging effect of fucoxanthin on DPPH inhibition as a percentage (%) was calculated as described by Hien et al. [41]. Ascorbic acid (at concentrations of 4, 20, and 100 μg mL^−1^) was used as the positive control. All experiments were performed in triplicate.

### 2.12. AChE Inhibitory Activity Assay

Inhibition of AChE activities by fucoxanthin was analyzed and calculated by using an Acetylcholinesterase Inhibitor Screening Kit (MAK324, Sigma, New Jersey, USA) according to the manufacturer’s instructions. Galantamine (at concentrations of 4, 20, 100, and 500 µg mL^−1^) was used as a positive control. AChE activity was evaluated based on the absorbance of fucoxanthin or galantamine measured at a wavelength of 412 nm, which was obtained by using a microplate reader (Thermo Fisher Scientific, Inc., Waltham, MA, USA). The results were given as IC_50_ [42], and all experiments were performed in triplicate.

### 2.13. Cell Culture and Treatment

C6 rat glial cells can be differentiated into disease-specific phenotypes, such as Alzheimer’s disease, by some factors, such as LPS, amyloid beta (Aβ), etc. [43,44]. Therefore, we use this cell line for our research.

C6 rat glial cells (ATCC, CCL-107™) were cultured in Dulbecco’s minimum essential medium (DMEM)/high glucose supplemented with 10% (*v*/*v*) fetal bovine serum (FBS) and 1% (*v*/*v*) penicillin/streptomycin under 5% CO_2_ at 37 °C.

For the cell viability test, C6 cells were cultured in DMEM/high glucose in a 96-well culture plate at a cell density of 0.5 × 10^5^ cells well^−1^ for 24 h. After that, the cells were incubated with fucoxanthin at different concentrations of 1, 10, and 100 μM for 24 h. Then, cell viability was determined by using a 3-(4,5-dimethylthiazol-2-yl)-2,5-diphenyltetrazolium bromide (MTT) assay.

To assess the protective ability against oxidative stress induced by H_2_O_2_ or fucoxanthin, C6 cells were cultured in DMEM/high glucose in 96-well culture plates at a density of 0.5 × 10^5^ cells well^−1^ for 24 h. After that, the cells were incubated with fucoxanthin at different concentrations of 1, 10, and 100 μM for 24 h or ascorbic acid (20 µg mL^−1^) as a positive control for another 24 h, followed by 1 h of incubation with H_2_O_2_ solution (10 mM). The cytoprotective effect of fucoxanthin samples against oxidative stress induced by H_2_O_2_ on C6 cells was indicated by the cell survival rate using the MTT assay method.

To assess the neuroprotection activity of fucoxanthin against Aβ_25–35_-induced cytotoxicity, C6 cells were cultured for 24 h in DMEM/high glucose in a 96-well culture plate at a density of 0.5 × 10^5^ cells well^−1^. After that, the cells were incubated with fucoxanthin at different concentrations of 1, 10, and 100 μM for 24 h or galantamine (0.1 µg mL^−1^; Sopharma AD, Bulgaria) as a positive control for another 24 h, followed by 24 h of incubation with protein Aβ_25–35_ (20 μM) [45]. The experiment consisted of the following groups: a control group (EtOH − Aβ_25–35_); model group (EtOH + Aβ_25–35_); and experimental group (fucoxanthin or galantamine + Aβ_25–35_). Each experiment was performed in triplicate. Cell viability was determined using the MTT assay method.

### 2.14. MTT Assay

The cell viability after treatment was determined through a MTT assay, and the cell survival rate was calculated as described by Hien et al. [41]. All experiments were performed in triplicate.

### 2.15. Measurement of Antioxidant Enzymes

C6 cells were incubated with fucoxanthin at different concentrations of 50 and 100 μM or ascorbic acid (20 µg mL^−1^) for 24 h. Afterward, the cells were washed twice with ice-cold phosphate buffered saline (PBS, pH 7.4). We collected the residue by centrifugation at 12,000× *g* for 5 min at 4 °C and discarded the supernatant. The pellet was ultrasonically extracted, and centrifuged at 12,000 rpm min^−1^ for 10 min at 4 °C to obtain the supernatant. The supernatant was used to identify the activity of the enzymes SOD, CAT, and glutathione peroxidase (GPx), as described by Weydert et al. [46]. The total amount of protein was determined by a Bradford protein assay (Bio-Rad, Hercules, CA, USA) for normalization [47].

### 2.16. Real-Time Polymerase Chain Reaction (qPCR)

Total RNA was extracted from C6 cells using TRIzol™ Reagent (Invitrogen, Singapore) according to the manufacturer’s instructions. Quantitative real-time PCR was performed in the MyGo Pro real-time PCR instrument (IT-IS Life Science Ltd., Dublin, Ireland) using a Luna^®^Universal One-Step RT-qPCR Kit (New England BioLabs Inc., Hertfordshire, UK). The primer sequences are shown in Table 2. The expression levels of target genes were normalized to that of β-actin by the normalization of expression (CT) method according to the guidelines of the manufacturer.

### 2.17. Statistical Analysis

Data are expressed as the mean ± standard error of the mean (SEM). Statistically significant differences were evaluated using Student’s *t*-test. Differences were considered statistically significant at *p* < 0.05.

## 3. Results

### 3.1. Screening Experiment to Identify Species of the Sargassum Genus with the Potential to Accumulate Fucoxanthin

A screening experiment was performed with nine seaweed species of the genus *Sargassum* that were rich in fucoxanthin and collected at different locations in Thua Thien Hue, Khanh Hoa, and Ninh Thuan, Vietnam, in 2007–2008 and 2022, as presented in Table 1. As shown in Table 3, various species of *Sargassum* can accumulate fucoxanthin, ranging from 3.82 to 2927.98 μg g^−1^ on a dry weight basis. *S. oligocystum* harvested from Khanh Hoa, Vietnam, during 15–17 Jan 2008 had the highest fucoxanthin accumulation capacity of 2927.98 μg g^−1^ on the dry weight biomass. Therefore, this seaweed species was selected to extract fucoxanthin for further studies.

*Sargassum* generally grows well from October to June each year. The harvest season of *Sargassum* species in Vietnam is from March to April and September to October each year. The results in Table 3 show that the fucoxanthin content in the *Sargassum* genus varied greatly by species, geographical location, and harvest time. Two species, *S. oligocystum* and *S. mucclurei*, showed high fucoxanthin contents of 2927.98 ± 8.01 and 1650.03 ± 7.10 µg g^−1^ on a dry weight basis, respectively. Therefore, we observed fluctuations in the fucoxanthin content of these two species of *Sargassum* according to the season of the year, and the data are presented in Table 4.

The results in Table 4 show that the fucoxanthin content of *S. oligocystum* was much higher than that of *S. mucclurei*. In December and January, the highest fucoxanthin content was recorded in *S. oligocystum* and *S. mucclurei*. In addition, both species are largely abundant in Vietnam and can be used as a good source of raw materials for fucoxanthin extraction. However, in samples from Vietnam, the fucoxanthin content of *S. oligocystum* species was much higher than that of *S. mucclurei*. This prompted us to extract a large amount of fucoxanthin from this *S. oligocystum* biomass for further studies.

### 3.2. Purification and Quantification of Fucoxanthin by Column Chromatography and Thin-Layer Chromatography

Until now, no publications have examined the fucoxanthin concentration from species of *S. oligocystum* worldwide. Crude fucoxanthin extracted from the biomass of Vietnam *S. oligocystum* was purified by column chromatography and TLC. The results are shown in Figure S1A, which shows that the fucoxanthin extracted from *S. oligocystum* species exhibits a similar band (wells 2, 3, 4) to the standard fucoxanthin (well 1). The silica gel containing these bands was further used for HPLC analysis. Through HPLC analysis, the fucoxanthin content was analyzed and quantified as 2562.78 ± 9.15 µg g^−1^ dry weight biomass. The HPLC profile of fucoxanthin from *S. oligocystum* is shown in Figure S1D,E.

### 3.3. Determining the Structure of the Isolated Compound

**Figure 1:**
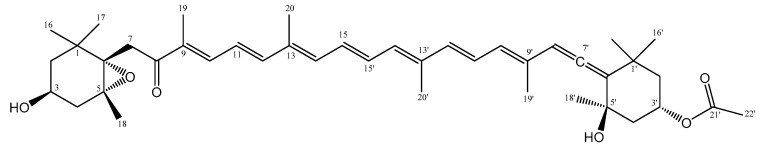
Chemical structure of extracted fucoxanthin isolated from Vietnamese *S. oligocystum*.

Compound-1 was isolated as red needles. The ^1^H NMR and ^13^C NMR spectra of compound-1 showed signals assignable to polyene possessing acetyl, conjugated ketone, two quaternary geminal dimethyls, two quaternary geminal methyls of oxygen, four olefinic methyls, and allene functionalities. These data suggested that compound-1 was a carotenoid, in which one hydroxyl group was acetylated (Figure 1 and Figure S1B,C).

^1^H NMR (CDCl_3_, 600 MHz) δ_H_ ppm: 0.96 (3H, s, H_3_-16), 1.03 (3H, s, H_3_-17), 1.07 (3H, s, H_3_-17′), 1.22 (3H, s, H_3_-18), 1.35 (1H, m, H-2ax), 1.36 (3H, s, H_3_-16′), 1.38 (3H, s, H_3_-18′), 1.42 (1H, m, H-2′ax), 1.49 (1H, t, *J* = 12.5 Hz, H-2eq), 1.50 (1H, m, H-4′ax), 1.78 (1H, m, H-4ax), 1.81 (3H, s, H_3_-19′), 1.94 (3H, s, H_3_-19), 1.99 (3H, s, H_3_-20), 1.99 (3H, s, H_3_-20′), 1.99 (1H, m, H-2′eq), 2.03 (3H, s, H_3_-22′), 2.28 (1H, m, H-4′eq), 2.32 (1H, m, H-4eq), 3.63 (1H, s, H-7a); 3.66 (1H, s, H-7b), 3.82 (1H, m, H-3), 5.38 (1H, m, H-3′), 6.05 (1H, s, H-8′), 6.13 (1H, d, *J* = 11.4 Hz, H-10′), 6.27 (1H, d, *J* = 11.4 Hz, H-14′), 6.35 (1H, d, *J* = 15.0 Hz, H-12′), 6.40 (1H, d, *J* = 11.4 Hz, H-14), 6.57 (1H, m, H-11), 6.59 (1H, m, H-11′), 6.63 (1H, m, H-15′), 6.65 (1H, d, *J* = 15.0 Hz, H-12), 6.76 (1H, d, *J* = 15.0 Hz, H-15), 7.14 (1H, d, *J* = 11.4 Hz, H-10).

^13^C NMR (CDCl_3_, 150 MHz) δ_C_ ppm: 11.82 (C-19), 12.75 (C-20), 12.90 (C-20′), 14.00 (C-19′), 21.15 (C-22′), 21.39 (C-18), 25.05 (C-17), 28.13 (C-16), 29.20 (C-18′), 31.25 (C-16′), 32.08 (C-17′), 35.16 (C-1), 35.77 (C-1′), 40.81 (C-7), 41.64 (C-4), 45.26 (C-4′), 45.45 (C-2′), 47.07 (C-2), 64.32 (C-3), 66.17 (C-5), 67.14 (C-6), 68.07 (C-3′), 72.69 (C-5′), 103.39 (C-8′), 117.52 (C-6′), 123.38 (C-11), 125.69 (C-11′), 128.54 (C-10′), 129.42 (C-15), 129.42 (C-14′), 132.51 (C-9′), 132.51 (C-15′), 134.54 (C-9), 135.42 (C-13), 136.63 (C-14), 137.11 (C-12′), 138.07 (C-13′), 139.10 (C-10), 145.04 (C-12), 170.49 (C-21′), 197.89 (C-8), 202.37 (C-7′).

### 3.4. Antioxidant Properties of Fucoxanthin 

In this study, fucoxanthin displayed significant antioxidant activities (Table 5). The percentage of radical scavenging activities in the DPPH assay of fucoxanthin at a concentration of 2 mg/mL was 31.80 ± 0.84% (Table 5). For the first time, the antioxidant activities of fucoxanthin isolated from Vietnamese *S. oligocystum* were reported in this study.

### 3.5. Acetylcholinesterase (AChE) Inhibitory Activity of Fucoxanthin 

Fucoxanthin showed AChE inhibitory activities with IC_50_ values of 130.12 ± 6.65 μg mL^−1^ (Table 6). Galantamine, a positive control, showed higher AChE inhibition, with an IC_50_ value of 1.78 ± 0.13 μg mL^−1^ compared to that of fucoxanthin (Table 6). The percentage of AChE inhibition of fucoxanthin extracted from *S. oligocystum* species at the tested concentration of 100 μg mL^−1^ reached 46.28 ± 1.05%, indicating that the extracts were moderate AChE inhibitors (with an IC_50_ value of 130.12 ± 6.65 µg mL^−1^). The AChE inhibition percentage of the positive control galantamine at a concentration of 10 μg mL^−1^ reached 87.39 ± 2.84% (with an IC_50_ value of 1.78 ± 0.13 μg mL^−1^).

### 3.6. Cytotoxic Effect of Fucoxanthon on C6 Cells

**Figure 2:**
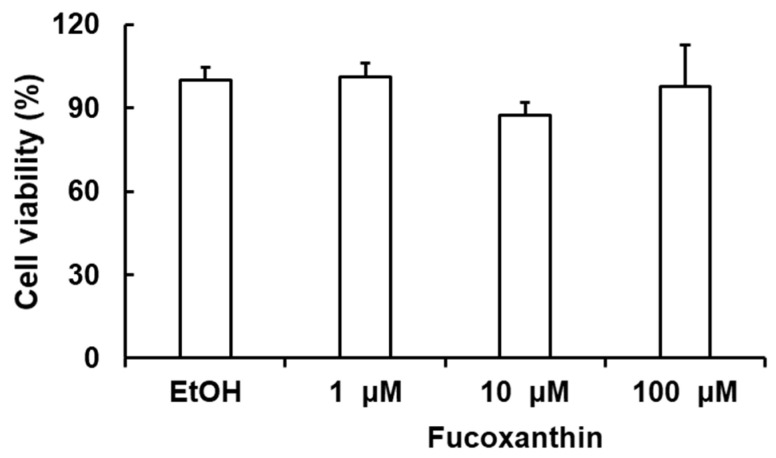
Effect of fucoxanthin on the survival of C6 cell lines. Ethanol (EtOH) was used as the control group. The data are expressed as the mean ± SD (*n* = 3).

To exclude that the toxicity of fucoxanthin exhibits secondary effects on neuroprotective activity, the cytotoxicity of this fucoxanthin was evaluated. The obtained results showed that the cell viability of C6 cells after incubation with fucoxanthin at concentrations of 1, 10, and 100 μM for 24 h was over 95%, suggesting that fucoxanthin was nontoxic at the tested concentrations (Figure 2).

### 3.7. Neuroprotective Effects of Fucoxanthin against Damage Caused by Oxidative Stress Induced by H_2_O_2_ on C6 Cell Lines

**Figure 3:**
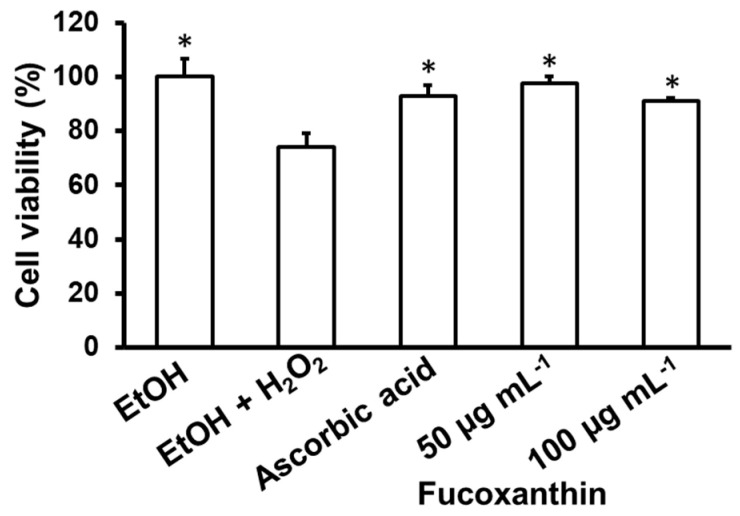
Neuroprotective effects of fucoxanthin against H_2_O_2_-induced oxidative stress in C6 cell lines. Cells were pre-incubated with fucoxanthin or ascorbic acid (20 µg mL^−1^) at the indicated concentrations for 24 h prior to 10 mM H_2_O_2_ exposure for 1 h. Cell viability was assessed by a MTT assay. The data are expressed as the mean ± SD (*n* = 3). Significant differences in the cell damage induced by H_2_O_2_ are denoted by * *p* < 0.05. EtOH: ethanol; H_2_O_2_: hydrogen peroxide.

In this study, treatment with 10 mM H_2_O_2_ significantly decreased cell viability by 74%, while this rate reached 100% when the cells were cultured in medium without H_2_O_2_ (in the ethanol (EtOH) group) (Figure 3). Preincubating the cells with ascorbic acid before adding the H_2_O_2_ supplement significantly inhibited the damaging effect of H_2_O_2_ on C6 cells. Similarly, pretreatment with fucoxanthin at concentrations of 50, 100, and 200 μg mL^−1^ protected C6 cells against H_2_O_2_-induced cell damage. The percentage of viability in cells pretreated with fucoxanthin at concentrations of 50 and 100 μg mL^−1^ increased to 97.69% and 91.23%, respectively, compared to that of cells treated only with H_2_O_2_ (Figure 3). Therefore, fucoxanthin at concentrations of 50 and 100 µg mL^−1^ was selected for the next experiment.

### 3.8. Fucoxanthin Protects C6 Cell Lines against Aβ_25–35_-Induced Cytotoxicity

**Figure 4:**
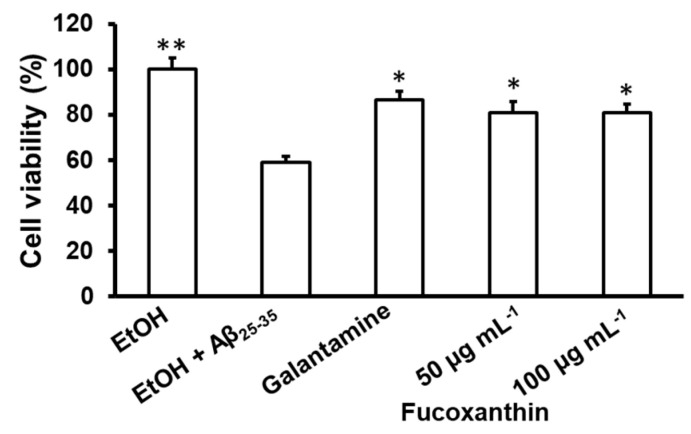
Neuroprotective effects of fucoxanthin against Aβ_25–35_-induced neurotoxicity in C6 cell lines. Cells were pre-incubated with fucoxanthin (at 50 and 100 µg mL^−1^) or galantamine (at 0.1 µg mL^−1^) for 24 h prior to 20 mM Aβ_25–35_ exposure for 1 h. Cell viability was assessed by MTT assay. The data are expressed as the mean ± SD (*n* = 3). Significant differences in the cell damage induced by Aβ_25–35_ are denoted by * *p* < 0.05; ** *p* < 0.01. EtOH: ethanol; H_2_O_2_: hydrogen peroxide.

Treatment with Aβ_25–35_ led to a decrease in cell viability from 100% in the control group to 59% in the Aβ_25–35_-treated group. However, pretreating C6 cells with fucoxanthin or galantamine (0.1 μg mL^−1^) before Aβ_25–35_ was added significantly attenuated Aβ_25–35_-induced cell death (Figure 4). At concentrations of 50 and 100 μg mL^−1^ of fucoxanthin, the cell survival rate significantly increased from 59.01% to 81.02% and 80.98%, respectively. The results indicate that fucoxanthin exhibits neuroprotective effects on C6 cells treated with Aβ_25–35_.

### 3.9. Cytoprotective Effects of Fucoxanthin on C6 Cell Lines against Damage by Oxidative Stress Induced by H_2_O_2_

**Figure 5:**
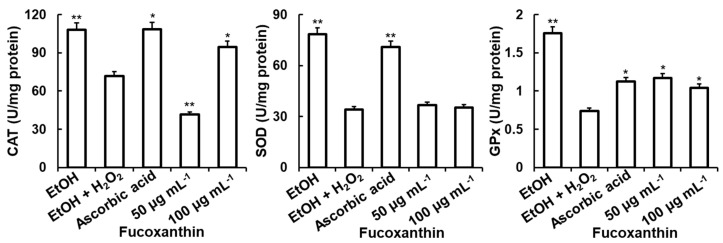
Effect of fucoxanthin on the activities of antioxidant enzymes in C6 cell lines. Cells were pre-incubated with fucoxanthin (concentrations of 50 and 100 µg mL^−1^) or ascorbic acid (20 µg mL^−1^) 24 h prior to 10 mM H_2_O_2_ exposure for 1 h. The data are expressed as the mean ± SD (*n* = 3). Significant differences in the cell damage induced by H_2_O_2_ are denoted by * *p* < 0.05; ** *p* < 0.001. SOD: superoxide dismutase; CAT: catalase; GPx: glutathione peroxidase; EtOH: ethanol; H_2_O_2_: hydrogen peroxide.

The results in Figure 5 show that ascorbic acid (concentration of 20 µg mL^−1^)-treated cells significantly increased the activity of SOD, CAT, and GPx by 107.47%, 51.22%, and 51.27%, respectively. Fucoxanthin significantly increased the activities of CAT and GPx and slightly induced the activity of SOD. Fucoxanthin at concentrations of 50 and 100 μg mL^−1^ increased GPx activities by 57.51% and 105.81%, respectively, compared to that of cells treated with H_2_O_2_ only. At a concentration of 50 μg mL^−1^, this fucoxanthin stimulation in C6 cells induced the activity of CAT by 31.98%.

### 3.10. Fucoxanthin Exhibits a Neuroprotective Effect by Regulating Genes Participating in Multiple Metabolic Pathways in C6 Cell Lines

**Figure 6:**
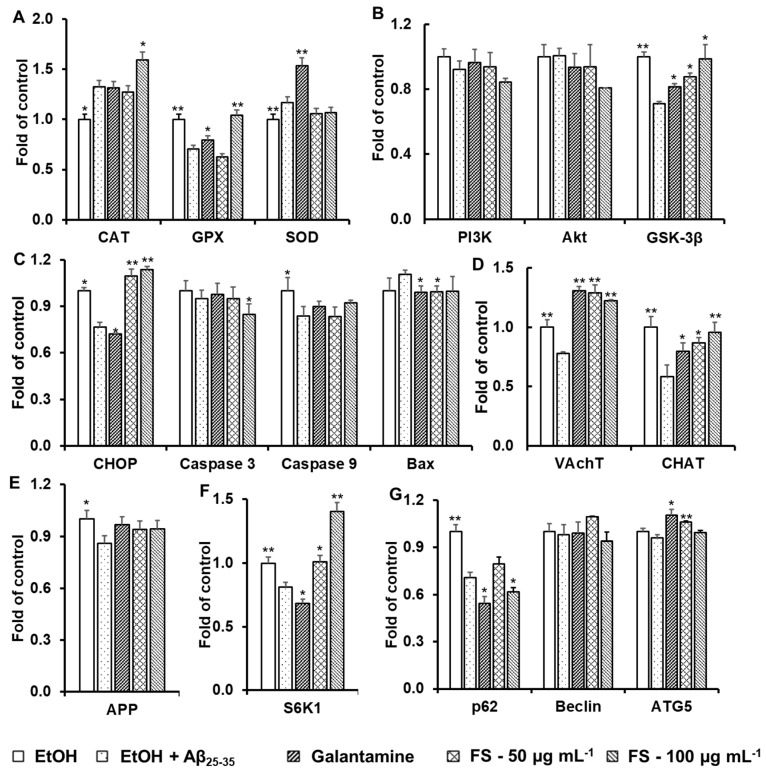
Effect of fucoxanthin on the expression of genes related to antioxidant enzymes (**A**), PI3K/Akt signaling (GSK-3ß) (**B**), the ER pathway (**C**), the biosynthesis of ACh (**D**), proteolytic processing (**E**), modulating protein translation (**F**), and autophagy (**G**) in C6 cell lines. The expression levels of genes were assessed with quantitative real-time PCR and normalized to β-actin in cells stimulated with fucoxanthin (concentrations of 50 and 100 µg mL^−1^) or galantamine (concentration of 0.1 μg mL^−1^) for 24 h prior to 20 mM Aβ_25–35_ exposure for 1 h. EtOH was used as control. The data are expressed as the mean ± SD (*n* = 3). Significant differences in the cell damage induced by Aβ_25–35_ are denoted by * *p* < 0.05; ** *p* < 0.001. EtOH: ethanol; *SOD*: superoxide dismutase; *CAT*: catalase; *GPx*: glutathione peroxidase; *PI3K*: phosphoinositide 3-kinase; *Akt*: protein kinase B; *GSK-3β*: glycogen synthase kinase 3β; *CHOP*: CCAAT/enhancer-binding protein homologous protein; *Bax*: Bcl2-associated X; *CHAT*: choline acetyltransferase; V*AChT*: vesicle acetylcholine transporter; *APP*: amyloid precursor protein; *S6K1*: S6 kinase 1; *p62*: sequestosome-1/A170/Zeta-interacting protein; *ATG 5*: autophagy-related gene 5.

The mRNA expression of antioxidant enzymes, such as SOD, CAT, and GPx, in C6 cell lines was observed, and the mRNA expression of the antioxidant enzymes CAT and GPx increased in fucoxanthin-incubated cells compared to the Aβ_25–35_-treated group. The results showed a similar trend with enzyme activities (Figure 6A).

In this study, we found that pretreatment with fucoxanthin significantly reversed the decrease in *GSK3β* induced by Aβ_25–35_, suggesting that fucoxanthin may protect against Aβ_25–35_-induced neuronal death by reversing the inhibition of the *PI3-K*/*Akt* cascade (Figure 6B). However, in this study, increased expression levels of *PI3K* and *Akt* were not detected in all tested samples (galantamine and fucoxanthin) compared to the H_2_O_2_-treated group (Figure 6B). Furthermore, the protective effect of fucoxanthin against Aβ_25–35_-induced apoptosis and ER stress in C6 cell lines was investigated. Moreover, fucoxanthin significantly reduced the expression levels of genes encoding apoptotic proteins, such as *caspase*-3 and *Bax* (Figure 6C). These results are consistent with previous results obtained by other authors, including Dhami et al. [59], Nisa et al. [60], and Sodik et al. [61]. 

Additionally, we found that Aβ_25–35_-treated C6 cells significantly suppressed the mRNA levels of *ChAT* and *VAChT* compared to the control sample. However, the values significantly improved when C6 cells were treated with galantamine and fucoxanthin (Figure 6D). Amyloid peptide (Aβ), generated by proteolytic cleavage of amyloid precursor protein (APP), plays an important role in the pathogenesis of Alzheimer’s disease (AD). Lin et al. [56] reported that curcumin reversed metal ion-induced AD in PC-12 cells by inhibiting excessive expression of *APP* and *BACE1*. In our study, fucoxanthin did not inhibit the expression level of APP compared to that in the Aβ_25–35_-treated group (Figure 6E).

We found that galantamine significantly inhibited the mRNA level of *S6K1*, while fucoxanthin exhibited an inverted effect (Figure 6F). Therefore, the neuroprotective effect of fucoxanthin was not mediated by modulating protein translation. Here, we observed low mRNA levels of *p*62 and a higher level of *ATG*5 (autophagy-related 5) in the fucoxanthin group than in the Aβ_25–35_-treated group (Figure 6G). Above all, the results showed that fucoxanthin isolated from *S. oligocystum* of Vietnam may exert a neuroprotective role in the AD process by stimulating autophagy and autophagic flux. 

## 4. Discussion

Numerous bioactive molecules found in *Sargassum* species have been studied to analyze their diverse pharmacological effects, such as antioxidant, anticancer, anti-inflammatory, antibacterial, anticoagulant, and neuroprotective activities [6]. Hence, we isolated and evaluated the bioactivities, especially the neuroprotective effect of fucoxanthin isolated from Vietnamese *S. oligocystum*, which could provide comprehensive insight into the chemical structures and biological activities. In addition, the results could provide information that could lead to the development of more different applications, especially applications in human health food based on *Sargassum*.

According to Terasaki et al. [62], the total fucoxanthin and lipid content of seaweed species varies with the location and season. Our obtained screening results (two species of *S. oligocystum* and *S. mucclurei* among the nine species studied contained high fucoxanthin contents of 2986.28 ± 9.01 and 1658.81 ± 7.17 µg g^−1^ for the dry weight biomass, respectively, as shown in Table 4) were consistent with a previous report from Jaswir et al. [63]. The fucoxanthin contents of *S. duplicatum* and *S. binderi* were reported to be 1010 µg g^−1^ and 730 µg g^−1^ dry weight, respectively. The fucoxanthin content of *S. angustifolium* (harvested from the Persian Gulf coast of Iran) was recorded to be 700 µg g^−1^ dry weight [64]. In Japan, the fucoxanthin content of *S. horneri* was reported to be 3700–10,810 µg g^−1^ dry weight when cultured in December to June at temperatures from 2.8 to 13.6 °C, and the highest levels of fucoxanthin were obtained in February (10,810 µg g^−1^ dry weight) [62].

For the first time in Vietnam, the fucoxanthin content of nine species of *Sarrgasusm* genera was identified in this study. Moreover, to date, no published report has measured the fucoxanthin content of *S. oligocystum* seaweed. Furthermore, the reserves of *S. oligocystum* seaweed in Vietnam are very large and contain high fucoxanthin content; thus, this seaweed can be exploited as a health food with neuroprotective biopharmaceutical effects from January to February every year.

Chemical structure identification was performed using NMR spectroscopy, which is an effective method for determining the structure of fucoxanthin. The NMR data of compound-1 (Figure 1) were in agreement with the previously reported literature [40,65]. Fucoxanthin was a potent antioxidant, as its chemical structure includes an epoxide group, hydroxyl group, and allenic bond [8]. Fucoxanthin isolated from seaweed exhibits an effective radical scavenging ability [66,67]. Here, we found that the radical scavenging activity of fucoxanthin (Table 5) was higher (8.34–31.80%) than that from *S. fusiforme* (24.3%), *S. marginatum* (11%), *S. myriocystum* (10–25%), and *S. pallidum* (29.4%) [68,69,70]. According to previous reports by Nisa et al. [60] and Sodik et al. [61], the antioxidant activity of fucoxanthin extracted from *S. filipendula* and *Sargassum* sp. was assessed by the DPPH method, with IC_50_ values of 0.639 and 0.087 mg mL^−1^, respectively, compared with our data in this study (IC_50_ value of 3.42 mg mL^−1^; Table 5). According to a publication by Savira et al. [71], the methanol extract of *S. duplicatum* showed the highest antioxidant activity, with an IC_50_ value of 78.52 ppm (78.52 μg mL^−1^), followed by the ethanol extract, with an IC_50_ value of 93.77 ppm (93.77 μg mL^−1^) and acetate value of 112.3 ppm (112.3 μg mL^−1^). Thus, the antioxidant activity of fucoxanthin isolated from Vietnamese *S. oligocystum* was lower than that reported in the above studies. This difference may be due to variations in *Sargassum* species from the collected region, seasonal variations, harvest time, solvent selection, and different extraction methods used for the study.

Kawee-ai et al. [72] reported that the crude extracts of *Phaeodactylum tricornutum* exhibited strong AChE inhibitory activities, with an IC_50_ value of 1.65 mg mL^−1^. Natarajan et al. [73] reported that the AChE inhibitory activities with IC_50_ values of extracts from *Gracilaria gracilis*, *Sargassum*, and *Cladophora fasicularis* were approximately 2 mg mL^−1^. The IC_50_ value of fucoxanthin showed high inhibitory activity against AChE up to 130.12 ± 6.65 µg mL^−1^ (or 0.130 mg mL^−1^; Table 6) compared with reports of Natarajan et al. [73] and Kawee-ai et al. [72]. We suggest that fucoxanthin in this study may be a useful approach for AD treatment and can be used in functional food applications for human health benefits.

According to Vinutha et al. [74], plant extracts/substances with strong or weak AChE inhibitory effects were classified based on their percentage of AChE inhibition, in which a weak inhibitor is below 30% inhibition; a moderate inhibitor is in the range of 30–50%; and a potential inhibitor is over 50%. Compared with the positive control galantamine, the IC_50_ value of fucoxanthin in this study was lower, reaching 130.12 ± 6.65 μg mL^−1^ (Table 6). Alghazwi et al. [4] discovered the effects of inhibiting AChE and butyrylcholinesterase (BChE), reducing oxidative stress, increasing anti-inflammatory activity, inhibiting kinase, enhancing neurodevelopment, and reducing the neurotoxicity of dopamine receptors of fucoxanthin extracted from seaweed in in vitro models. Lin et al. [75] also reported that fucoxanthin extracted from *S. horneri* inhibited AChE activity, with an IC_50_ value of 81.2 µM (equivalent to 53.43 μg mL^−1^). The results we obtained for fucoxanthin extracted from Vietnamese *S. oligocystum* are also similar to the above studies (graded as a moderate inhibitor of AChE).

Cell damage induced by oxidative stress factors plays an important role in the development of neurodegenerative diseases, such as AD [76]. Studies have shown that oxidative stress induced by H_2_O_2_ leads to peroxidation of membrane lipids and cell death [77]. The neuroprotective effects of fucoxanthin are comparable to those of ascorbic acid, and other studies published by Xiang et al. [78] on the SH-SY5Y cell line as a model for AD and Alghazwi et al. [79] on PC-12 cells suggest that treating C6 cells with fucoxanthin inhibits H_2_O_2_-induced oxidative stress.

Heo et al. [80] found that fucoxanthin isolated from *S. siliquastrum* at concentrations of 5–200 µM reduced the toxicity induced by H_2_O_2_ in Vero cells, while Alghazwi et al. [79] suggested that pretreatment with fucoxanthin (0.1–2 µM) increased the survival rate of cells damaged by Aβ_25–35_ up to 67–98.5%. Choi et al. [81] also indicated that an alcohol extract of *S. serratifoilum* reduced the accumulation of Aβ_1–42_ in Chinese hamster ovary cells (CHO-751 cells). Our results (Figure 3 and Figure 4) correspond with the statement that concentrations of the tested sample likely depend on cell lines, and some cell lines, such as PC-12 cells, are more sensitive than others.

In the presence of ochratoxin A (mycotoxin), SOD, CAT, and GPx, crucial antioxidant enzymes, act as endogenous antioxidants against free radicals and eliminate excessive free radicals produced in cells by directly catalyzing the disintegration process of H_2_O_2_ to O_2_ under oxidative stress [82]. Several lines of evidence have indicated that fucoxanthin (isolated from edible brown algae or from *S. horneri*) provides neuroprotection against H_2_O_2_ and Aβ oligomer-induced apoptosis and oxidative stress in cell lines via mechanisms that involve the improvement of antioxidant enzyme activities, the activation of the PI3K/Akt pathway, and the inhibition of the ERK pathway [29,83,84]. The results of our study with FS are shown in Figure 5 and Figure 6A,B, with similar results to the above fucoxanthin isolated from edible brown algae or from *S. horneri*, respectively, reported by Yang et al. (2021b) [83], Lin et al. (2017) [29], and Yu et al. (2017) [84].

Exposure of cells to Aβ oligomers or H_2_O_2_ leads to apoptotic neuronal cell death by increasing oxidative stress, possibly as a result of altered regulation of signaling pathways [85,86,87]. In neurons, these factors substantially increase the consumption of oxygen and the production of intracellular reactive oxygen species (ROS) [87]. Moreover, Aβ oligomers and H_2_O_2_ were reported to induce neuronal apoptosis by inhibiting the prosurvival phosphoinositide 3-kinase (PI3K)/Akt signaling pathway and overactivating the downstream glycogen synthase kinase 3β (GSK-3β) in vitro [85]. According to Yu et al. [84] and Lin et al. [29], fucoxanthin (isolated from edible brown algae and from *S. horneri*) exhibits an inhibitory effect against H_2_O_2_- and Aβ oligomer-induced cell toxicity in SH-SY5Y cells. This process is activated by the PI3K/Akt cascade and inhibition of the ERK pathway. However, in our study, we did not observe an increase in the expression levels of *PI3K* and *Akt* in all tested samples (galantamine and fucoxanthin isolated from *S. oligocystum* of Vietnam) compared to H_2_O_2_ (Figure 6B). This occurred because the authors determined changes in protein content using an antibody, rather than at the expression levels of genes encoding these proteins. In the next study, we may need to confirm how fucoxanthin (isolated from *S. oligocystum* of Vietnam) alters the activation of the Akt/Nrf2/ARE pathway via protein expression levels.

The Aβ_25–35_ protein exerts many negative effects on cells, such as altered mitochondrial and endoplasmic reticulum (ER) morphology, increased Aβ aggregation, cell viability loss and apoptosis, cytochrome c release from mitochondria, ROS production, and intracellular calcium elevation [23,24,25]. In this study, the protective effect of fucoxanthin against Aβ_25–35_-induced apoptosis and ER stress in C6 cell lines was investigated (Figure 6C). *CHOP* and *caspase*-12 play an important role in ER stress-mediated apoptosis. They are activated to promote the apoptosis signaling pathway when cellular stress is very serious or prolonged [88]. It has been demonstrated that brain ischemia or hypoxia leads to increased expression of *CHOP* mRNA, neuronal apoptosis, and oxidative damage [89]. Normally, *caspase-*12 occurs in an inactive pro-enzyme state and is attached to the ER membrane [90]. Once it is activated, *caspase*-12 triggers *caspase*-3, *caspase*-9, and *Bcl*2-associated X (Bax) activation, resulting in DNA fragmentation.

Choline acetyltransferase (ChAT) is synthesized in the perikaryon of cholinergic neurons and is involved in ACh biosynthesis. Fujii et al. [91] showed that altering the expression of ChAT causes the ACh content in cells to be induced or reduced. The synthesis, storage, and release of acetylcholine require the expression of several specialized proteins, including ChAT and the vesicular ACh transporter (VAChT). The *VAChT* gene is within the first intron of the *ChAT* gene, and this structure helps activate the expression of the two genes by extracellular factors. A cholinergic deficit is among the primary features of AD, which is also characterized by amyloid deposition, and it could be attributed to the suppression of cholinergic markers in the absence of cell death; however, little is known about factors that reduce the expression of cholinergic phenotypes [92]. Our findings shown in Figure 6D were similar to those of previous studies [78], in which fucoxanthin reversed the reduction in Aβ oligomer-induced ChAT in the hippocampal regions and reduced the loss of cholinergic neurons caused by Aβ oligomers.

Our results shown in Figure 6E indicated that multiple mechanisms, including the activation of S6 kinase 1 (*S6K*1), which directly phosphorylates tau, may be directly involved in AD. Therefore, inhibiting *S6K*1 reduces the expression of *BACE*1 (which cleaves amyloid precursor protein to initiate the β-amyloid pathway) and tau [52].

Several studies have demonstrated that abnormal autophagy leads to the pathological state of AD [30]. In early AD, autophagy is strongly promoted, resulting in an increase in the clearance of misfolded proteins and neuronal viability, while the damaged autophagy function leads to the inhibition of impaired protein aggregation and suppresses cell survival in AD progression [93]. Autophagy is promoted by a number of key autophagy-related genes, such as *Beclin*-1, autophagy-related gene (*ATG*)5, *ATG*12, and *LAMP*-1.

Based on our results, the neuroprotective effects of fucoxanthin vary among samples isolated from different sources. In particular, fucoxanthin isolated from *S. oligocystum* in Vietnam protected against H_2_O_2_- and Aβ_25–35_-induced neurotoxicity in C6 cell lines by regulating the gene expression of antioxidant enzymes (*CAT* and *GPx*) (Figure 6A) and the ER pathway (*caspase*-3 and *Bax)* (Figure 6C). Fucoxanthin isolated from *S. oligocystum* in Vietnam also promoted the expression of a gene(s) involved in PI3K/Akt signaling (*GSK*-3β) (Figure 6B), autophagy (*p*62 and A*TG*5) (Figure 6E), and the biosynthesis of ACh (*VAChT* and *ChAT*) (Figure 6G). Thus, to date, no publication has examined fucoxanthin extracted from *S. oligocystum* seaweed. Our results provide the first report on the neuroprotective activity of fucoxanthin extracted from the brown Vietnamese seaweed *S. oligocystum*, providing a scientific basis for exploiting *S. oligocystum* seaweed, which is naturally abundant and provides a functional and healthy food for Vietnamese people, especially those who work in arduous conditions.

## 5. Conclusions

Among nine species of Vietnamese *Sargassum*, *S. oligocystum* Montagne, 1845 was found to produce the highest fucoxanthin content of 2986.28 µg g^−1^ on a dry weight basis. Fucoxanthin was isolated, purified, and structurally identified from Vietnamese *S. oligocystum* by various chromatographic techniques, including NMR. The fucoxanthin extracted from Vietnamese *S. oligocystum* exhibited AChE inhibitory and DPPH free radical scavenging activities. The IC_50_ values for DPPH-radical and AChE-inhibitory-activity of fucoxanthin were found to be 3.42 ± 0.15 mg mL^−1^ and 130.12 ± 6.65 µg mL^−1^, respectively, compared to 19.43 ± 0.13 μg mL^−1^ and 1.78 ± 0.13 μg mL^−1^ for the reference drugs ascorbic acid and galantamine, respectively. This fucoxanthin showed no cytotoxicity against C6 cell lines up to 100 μM and exhibited more powerful cytoprotective effects on H_2_O_2_- and Aβ_25–35_-induced neurotoxic C6 cell damage at concentrations of 50 and 100 µg mL^−1^. Fucoxanthin isolated from *S. oligocystum* of Vietnam exhibited a neuroprotective effect by protecting against H_2_O_2_- and Aβ_25–35_-induced neurotoxicity in C6 cell lines; fucoxanthin achieved this effect by regulating the activity and gene expression of antioxidant enzymes (such as *CAT* and *GPx*) and the ER pathway (such as *caspase*-3 and *Bax*), as well as promoting the expression of genes involved in PI3K/Akt signaling (*GSK*-3β), autophagy (*p*62 and *ATG*5), and the biosynthesis of acetylcholine (*VACh*T and *ChAT*). The present results showed that fucoxanthin isolated from Vietnamese *S. oligocystum* has potential for applications in health foods and in the prevention and treatment of AD in Vietnam.

## Figures and Tables

**Table 1:** List of *Sargassum* species and their scientific names, including the places and times for the samples collected in this study.

Number	Scientific Name	Places and Times for Collecting	With Coordinates
1	*Sargassum mcclurei* Setchell, 1933	Hon Chong, Nha Trang, Khanh Hoa province; 20–21 September 2007, 2008; 27 April 2022 and 19 July 2022.	12°16′ N 109°12′ E
2	*S. binderi* Sonder ex J. Agardh, 1848	Bau Hamlet, Vinh Nguyen, Nha Trang; 15–20 October 2007	12°13′ N 109°14′ E
3	*S. polycystum* C. Agardh, 1824	My Hoa, Ninh Hai, Ninh Thuan province; 15–20 October 2007	11°42′ N 109°12′ E
4	*S. duplicatum* Bory	Hon Chong, Vinh Phuoc, Nha Trang, Khanh Hoa province; 15–20 October 2007	12°16′ N 109°12′ E
5	*S. denticarpum* T. Ajusaka, 1994	My Hoa, Ninh Hai, Ninh Thuan province; 15–20 October 2007	11°42′ N 109°12′ E
6	*S. swartzii* (Turn.) C. Ag.	Thua Thien Hue province; 15–20 October 2007	16°32′ N 107°39′ E
7	*S. microcystum* J. Agardh, 1848	Tri Nguyen Island, Nha Trang, Khanh Hoa province; 20–30 October 2007	12°11′ N 109°13′ E
8	*S. crassifolium* J. Agardh	Bai Tien, Vinh Hoa, Nha Trang, Khanh Hoa province; 20–30 October 2007	12°13′ N 109°12′ E
9	*S. oligocystum* Montagne, 1845	Hon Chong, Nha Trang, Khanh Hoa province; 20–21 September 2007; 2008; 27 April 2022; 19 July 2022	12°16′ N 109°12′ E

**Table 2:** Primer sequences for real-time PCR.

Primer	Sequences (5′-3′)		References
	Forward	Reverse	
CHAT	AGCCCTGCTGTGATCTTTGCTCG	CCTTGGCCCAGTCAGTGGGAA	[48]
VAChT	CCCTTAAGCGGGCCTTTCATTGAT	AAAGGCAAACATGACTGTGGAGGC	[48]
SOD	GCCTGGATGGCTACGTACA	GGTCCAGCGGATGAAGAG	[49]
CAT	AATGAAGACAACGTCACTCAGG	TGTTCTCACACAGGCGTTTC	[49]
GPX	GCAATCAGTTCGGACATCAG	CACCGGGTCGGACATACTT	[49]
PI3K	AACACAGAAGACCAATACTC	TTCGCCATCTACCACTAC	[50]
Akt	GTGGCAAGATGTGTATGAG	CTGGCTGAGTAGGAGAAC	[50]
GSK-3β	CATCCTTATCCCTCCTCACGCT	TATTGGTCTGTCCACGGTCTCC	[51]
S6K1	CTCTGAGGATGAGCTGGAGG	TTCTCACAATGTTCCATGCC	[52]
CHOP	GGAGAAGGAGCAGGAGAATGA	AGACAGACAGGAGGTGATGC	[53]
Atg5	CCCTGAAGACGGAGAGAAGA	TGCTGATGTGAAGGAAGTTGTC	[54]
p62	CCTATTACCTGGCCTGTGGA	GTTCATCCGTTGTGCATGAG	[54]
Caspase-3	GTGGAACTGACGATGATATGGC	CGCAAAGTGACTGGATGAACC	[55]
APP	GGACGACTCCGATGTCTGGT	ACATCAAAGTACCAGCGGGAG	[56]
Caspase-9	AGCCAGATGCTGTCCCATAC	CAGGAGACAAAACCTGGGAA	[57]
Beclin	TGGAAATCACTCGTATCTGGAG	CCACCTCTTCTTTGAACTGCT	[54]
Bax	GCAGGGAGGATGGCTGGGGAG	TCCAGACAAGCAGCCGCTCACG	[58]
β-actin	CTAAGGCCAACCGTGAAAAG	GCCTGGATGGCTACGTACA	[49]

**Table 3:** Fucoxanthin content in the different species of *Sargassum* collected from Thua Thien Hue, Khanh Hoa, and Ninh Thuan provinces, Vietnam (2007–2008).

No.	Species Name	Place and Time of Samples Collection	Fucoxanthin Content (μg g^−1^ Dry Weight)
1	*S. mucclurei*	Hon Chong, Nha Trang, Khanh Hoa province, 5–10 December 2007	1650.03 ± 7.10
2	*S. binderi*	Vinh Nguyen, Nha Trang, Khanh Hoa province, 15–20 October 2007	296.07 ± 5.50
3	*S. polycystum*	My Hao, Ninh Hai, Ninh Thuan province, 15–20 October 2007	3.82 ± 0.89
4	*S. duplicatum*	Hon Chong, Nha Trang, Khanh Hoa province, 15–20 October 2008	255.03 ± 5.71
5	*S. denticarpum*	My Hoa, Ninh Hai, Ninh Thuan province, 15–20 October 2007	24.25 ± 2.72
6	*S. swartzii*	Thua Thien Hue province, 15–25 October 2007	217.60 ± 5.04
7	*S. microcystum*	Dao Tri Nguyen, Nha Trang Bay, Khanh Hoa province, 20–30 October 2007	161.52 ± 2.90
8	*S. crassifolium*	Bai Tien, Nha Trang, Khanh Hoa province, 20–30 October 2007	26.65 ± 2.86
9	*S. oligocystum*	Xom Bau, Vinh Nguyen, Nha Trang, Khanh Hoa province, 17–18 January 2008	2927.98 ± 8.01

**Table 4:** Fucoxanthin content from *S. oligocystum* and *S. mucclurei* in 2007 and 2008.

Sample Collection Time	*S. mucclurei* (µg g^−1^ Dry Weight)	*S. oligocystum* (µg g^−1^ Dry Weight)
21 September 2007	697.65 ± 5.78	978.22 ± 7.18
22 October 2007	897.82 ± 7.98	1362.82 ± 7.92
20 November 2007	1298.67 ± 6.71	1872.16 ± 8.62
20 December 2007	1658.81 ± 7.17	2276.19 ± 8.68
20 January 2008	1310.72 ± 7.81	2986.28 ± 9.01
20 February 2008	1259.17 ± 7.42	1968.82 ± 7.62
15 March 2008	1087.16 ± 6.72	1276.92 ± 6.73
17 April 2008	962.19 ± 5.17	872.19 ± 5.12
11 May 2008	802.29 ± 7.82	776.97 ± 5.89
25 June 2008	779.78 ± 5.82	672.08 ± 4.82
20 July 2008	563.74 ± 4.17	517.81 ± 4.78
10 August 2008	392.81 ± 4.12	439.78 ± 3.36

**Table 5:** Antioxidant activities of fucoxanthin.

DPPH Scavenging Activity (%)		DPPH Scavenging Activity (%)	
Concentrations (mg mL^−1^)	Fucoxanthin	Concentrations (μg mL^−1^)	Ascorbic Acid
0.1	8.34 ± 0.14	4	32.70 ± 0.52
0.4	13.97 ± 0.10	20	89.90 ± 1.31
2	31.80 ± 0.84	100	92.3 ± 1.83
IC_50_ (mg mL^−1^)	3.42 ± 0.15	IC_50_ (μg mL^−1^)	19.43 ± 0.13

Notes: Ascorbic acid: positive control; Fucoxanthin isolated from *S. oligocystum*; Antioxidant activity of fucoxanthin and ascorbic acid determined by DPPH method.

**Table 6:** Acetylcholinesterase inhibitory activities of fucoxanthin.

AChE Inhibition Activity (%)		AChE Inhibition Activity (%)	
Concentrations (µg mL^−1^)	Fucoxanthin	Concentrations (µg mL^−1^)	Galantamine
4	4.25 ± 0.10	0.08	9.51 ± 0.52
20	21.35 ± 0.84	0.4	25.72 ± 1.31
100	46.28 ± 1.05	2	53.02 ± 1.10
500	75.43 ± 1.46	10	87.39 ± 2.84
IC_50_ (µg mL^−1^)	130.12 ± 6.65	IC_50_ (µg mL^−1^)	1.78 ± 0.13

Notes: Acetylcholinesterase (AChE); Galantamine: positive control; Fucoxanthin: fucoxanthin isolated from Vietnamese *S. oligocystum*.

## Data Availability

Data are contained within the article.

## Supplementary Materials

The following supporting information can be downloaded at: https://www.mdpi.com/article/10.3390/biomedicines11082310/s1, Figure S1: Separation of fucoxanthin from *S. oligocystum* by thin layer chromatography (A). Standard fucoxanthin (well 1, with a concentration of 50 µg mL^−1^), extracts obtained in the methanol phase (well 2), extracts obtained in the acetone phase (well 3), and fucoxanthin obtained after purification of the crude fucoxanthin by column chromatography (well 4). The 1H NMR spectrum (B); The 13C-DEPT-NMR spectrum (C); HPLC chromatograms of standard fucoxanthin (D) and fucoxanthin isolated from Vietnamese *S. oligocystum* (E).
