# Supplementary material for: Isolation of Fucoxanthin from Sargassum oligocystum Montagne, 1845 Seaweed in Vietnam and Its Neuroprotective Activity
Source: Biomedicines. 2023 Aug 19;11(8):2310. doi: 10.3390/biomedicines11082310 (PMC10452663; doi:10.3390/biomedicines11082310)
Supplement: Supplementary file 1 [file biomedicines-11-02310-s001.zip › biomedicines-2538496-supplementary.pdf]

Supplementary figures

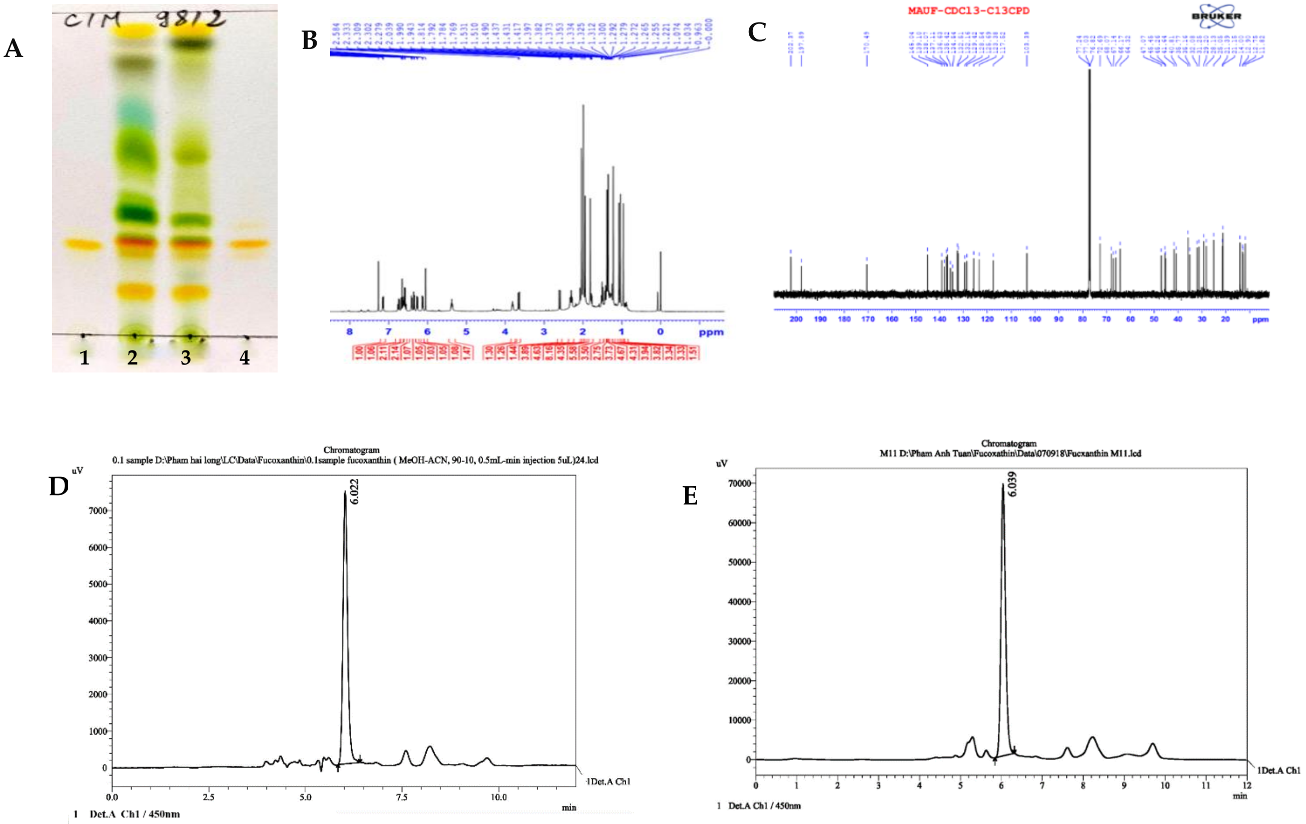

Figure S1. Separation of fucoxanthin from *S. oligocystum* by thin layer chromatography (A). Standard fucoxanthin (well 1, with a concentration of 50 µg mL^{-1}), extracts obtained in the methanol phase (well 2), extracts obtained in the acetone phase (well 3), and fucoxanthin obtained after purification of the crude fucoxanthin by column chromatography (well 4). The ^{1}H NMR spectrum (B); The ^{13}C-DEPT-NMR spectrum (C); HPLC chromatograms of standard fucoxanthin (D) and fucoxanthin isolated from Vietnamese *S. oligocystum* (E).
